# Biosynthesis of Polyhydroxyalkanoates in *Cupriavidus necator* B-10646 on Saturated Fatty Acids

**DOI:** 10.3390/polym16091294

**Published:** 2024-05-05

**Authors:** Natalia O. Zhila, Kristina Yu. Sapozhnikova, Evgeniy G. Kiselev, Ekaterina I. Shishatskaya, Tatiana G. Volova

**Affiliations:** 1 Institute of Biophysics SB RAS, Federal Research Center “Krasnoyarsk Science Center SB RAS”, 50/50 Akademgorodok, Krasnoyarsk 660036, Russia; kristina.sap@list.ru (K.Y.S.); evgeniygek@gmail.com (E.G.K.); shishatskaya@inbox.ru (E.I.S.); volova45@mail.ru (T.G.V.); 2 Basic Department of Biotechnology, School of Fundamental Biology and Biotechnology, Siberian Federal University, 79 Svobodnyi Av., Krasnoyarsk 660041, Russia

**Keywords:** degradable polyhydroxyalkanoates, PHAs, fatty acids, fatty acid mixture, biosynthesis, properties

## Abstract

It has been established that the wild-type *Cupriavidus necator* B-10646 strain uses saturated fatty acids (SFAs) for growth and polyhydroxyalkanoate (PHA) synthesis. It uses lauric (12:0), myristic (14:0), palmitic (16:0) and stearic (18:0) acids as carbon sources; moreover, the elongation of the C-chain negatively affects the biomass and PHA yields. When bacteria grow on C12 and C14 fatty acids, the total biomass and PHA yields are comparable up to 7.5 g/L and 75%, respectively, which twice exceed the values that occur on longer C16 and C18 acids. Regardless of the type of SFAs, bacteria synthesize poly(3-hydroxybutyrate), which have a reduced crystallinity (C_x_ from 40 to 57%) and a molecular weight typical for poly(3-hydroxybutyrate) (P(3HB)) (M_w_ from 289 to 465 kDa), and obtained polymer samples demonstrate melting and degradation temperatures with a gap of about 100 °C. The ability of bacteria to assimilate SFAs opens up the possibility of attracting the synthesis of PHAs on complex fat-containing substrates, including waste.

## 1. Introduction

The global production of synthetic, non-degradable plastics has reached 400 million tons per year [1,2]. The widespread accumulation of plastic waste is polluting the world’s oceans, thereby negatively affecting water quality and threatening biota [3,4]. Even more worrying is the increasing accumulation of microplastics in the biosphere [5]. Solving the problem of plastic waste is associated with a gradual transition to a new generation of degradable polymer materials [6,7], among which a special place belongs to polymers of microbiological origin, such as polyhydroxyalkanoates (PHAs).

PHAs are a family of biodegradable thermoplastic polymers of different chemical structures with various physicochemical properties [8,9,10,11]. At present, these biopolymers are rightfully considered as the most promising material of the 21st century for a wide variety of applications—ranging from the municipal and agricultural to pharmacology and biomedicine [12,13,14,15]. 

The key problem for increasing production volumes and expanding the scope of PHA application is reducing their cost, which is often achieved primarily through the use of the available carbon raw materials including waste. Potential raw materials for PHA synthesis can be various individual compounds, as well as, most importantly, in various types of waste (e.g., the products of processing and hydrolysis of plant materials, waste from food, pharmaceutical waste, alcohol waste, pulp and paper waste, etc.) [16,17,18,19,20,21].

The possibility of PHA synthesis from the waste of various origins is of great importance as it contributes to “The Circular Economy” [11,22,23,24]. The use of waste as substrates in biotechnological processes for obtaining target products is not only a way to reduce the volume of their accumulation in the biosphere, but also a way through which to increase the efficiency of industrial production and the complete use of raw materials in general [25].

Great potential is represented by fat-containing wastes, which are accumulated in huge quantities in the food industry due to the lack of rational and effective technologies for their processing. The amount of fat waste generated annually is about 29 million tons [26]. The waste of fat-containing raw materials, which are low-grade oils, waste cooking oils, as well as fish processing waste containing smoking components (phenolic compounds, polycyclic aromatic hydrocarbons, etc.) are unsuitable not only for humans, but also for use in animal and aquaculture feed. These wastes must be treated to prevent environmental pollution, or they should be disposed safely [27]. Such wastes often are transported to municipal solid waste landfills and burned, so the possibility of their use is a relevant solution. An environmentally significant area for the use of large-capacity fat waste can be the biotechnological synthesis of target products including degradable PHAs.

Despite the fact that interest in lipid substrates for PHA synthesis has emerged relatively recently, the results obtained are encouraging. The possibility of conducting PHA synthesis using vegetable oils of various origins [28], as well as the low-quality and waste fats of animal origin [29,30,31] and fat-containing fish processing waste [32,33,34,35], have been shown. It is important to note that the high-energy efficiency of the transformation of lipid substrates in microbial metabolism processes with a possible theoretical PHA yield is up to 0.7–0.8 g/g. This is practically twice as high when compared to the results for sugars [36,37,38].

However, a complicating factor in the synthesis of PHAs on complex fatty substrates may be the found in the selective consumption of fatty acids (FAs) by bacteria. Thus, during the PHA accumulation on complex fatty substrates, the uneven consumption of saturated, polyenoic and unsaturated FAs as a component of the C-substrate formed under the action of lipolytic enzymes was revealed [39]. 

The effect of uneven FA consumption was discovered in the culture of *Cupriavidus necator* B-10646 when the PHA synthesis on vegetable oils (sunflower and Siberian seed oils) in contrast to palm oil (the FA from which was utilized by bacteria evenly) was studied [40]. The aforementioned paper [41] showed that the *Ralstonia eutropha* H16 strain more actively metabolized palmitic, oleic and linoleic fatty acids when it was grown on soybean oil in contrast to linolenic acid, which was practically not used. When *C. necator* B-10646 was grown on the fish processing fatty waste obtained during the production of canned sprats and on the fat obtained from the heads and ridges of Atlantic mackerel, the bacteria primarily utilized polyenoic fatty acids (linoleic and linolenic acids) in contrast to saturated fatty acids and unsaturated monoenoic oleic acid, the content of which increased in the culture (which led to changes in the residual lipid saturation [42]). These results are comparable with the data of paper [43], which also noted the uneven consumption of FAs by bacteria during the PHA synthesis on pollock waste by six strains of *Pseudomonas*. The authors showed that these changes manifest themselves differently depending on the strain specificity of the bacteria, the synthesis route and PHA composition. Uneven FA utilization by bacteria and the accumulation of non-recyclable acids in the culture negatively affects the complete use of the substrate, thereby reducing the final yield of the product and the economics of the process as a whole.

At the same time, it is known that PHA producers metabolize many FAs as the sole carbon source, and their metabolism is associated with intracellular PHA synthesis. 

The potential of fatty acids as a substrate for PHA synthesis by representatives of various taxa is illustrated in Table 1. The results were obtained under the same conditions of laboratory cultivation of PHA producers in glass flasks as a fermentation vessel, which allows for a comparison of the achieved indicators. 

The most representative array of data was obtained from the study of the *Pseudomonas* strains, as well as the *Aeromonas hydrophila* [45,46,52], *Delftia* [49,53], *Burkholderia* sp. [50], *Klebsiella pneumonia* [45], *Bacillus cereus* [47], and *Rhodococcus pyridinivorans* [51] strains. There is very limited information regarding the ability of *Cupriavidus* strains to synthesize the PHAs on fatty acids (Table 1). The bacteria belonging to the genus *Cupriavidus* (formerly *Hydrogenomonas*, *Alcaligenes*, *Ralstonia*, and *Wautersia*) are promising producers of PHAs [54], both those under autotrophic conditions and on various organic substrates, including individual fatty acids [44,48,55,56]. The highly productive *Cupriavidus necator* B-10646 strain, which has broad organotrophic potential, is capable of synthesizing PHAs in high yields using mixtures of CO_2_ and H_2_ [57], sugars [58,59], and glycerol [60]. Regarding the ability of the *C. necator* B-10646 strain to synthesize PHAs, it has been mostly oleic acid that has been studied; moreover, the possibility of poly(3-hydroxybutyrate) and copolymers with a 3-hydroxyvalerate synthesis and the addition of valerate precursors to a medium has been shown [61,62]. It has also been shown that saturated fatty acids, as well as oleic acid, are poorly utilized by this strain when it is grown on complex fatty substrates [41,42].

The purpose of this work is to study the ability of the wild-type *Cupriavidus necator* B-10646 strain to metabolize saturated fatty acids as the sole carbon source for the synthesis of PHAs and the influence of the type of FAs on the composition and properties of polymers.

## 2. Materials and Methods

### 2.1. The PHA Producer Strain, Media and Cultivation Technique

In all experiments, the wild-type *Cupriavidus necator* B-10646 strain was used. It is registered in the Russian National Collection of Industrial Microorganisms (VKPM) [63]. It is able to synthesize PHAs using a wide range of carbon substrates (CO_2_, sugars, glycerol, plant oils, etc.).

The nutrient medium used was Schlegel’s mineral salt medium [64], which is based on a phosphate buffer (Na_2_HPO_4_, 9.1 g/L; KH_2_PO_4_, 1.5 g/L) containing a source of magnesium (MgSO_4_, 0.2 g/L), iron (Fe_3_C_6_H_5_O_7_, 0.025 g/L), nitrogen source (NH_4_Cl, 0.7 g/L) and a set of microelements (3 mL solution/L of medium; solution composition (g/L): H_3_BO_3_ (0.228), CoCl_2_ × 6H_2_O (0.03), CuSO_4_ × 5H_2_O (0.008), MnCl_2_ × 4H_2_O (0.008), ZnSO_4_ × 7H_2_O (0.176), NaMoO_4_ × 2H_2_O (0.05), NiCl (0.008)). The carbon sources were constituted of the following: saturated fatty acids (lauric (C12:0), myristic (C14:0), palmitic (C16:0) and stearic (C18:0) (purity 97%, Acros Organics, Brussels, Belgium), monounsaturated oleic acid (18:1) (purity 98%, EKOS-1, Staraya Kupavna, Russia) and mixtures thereof were used, as described in the text.

Bacteria were grown under aerobic conditions at a temperature of 30 °C in a thermostatic incubator shaker “Incubator Shaker Innova“ (New Brunswick Scientific, Edison, NJ, USA) in 0.5 L flasks. The bacteria were cultured in batch mode and in the conditions developed earlier for PHA biosynthesis. To obtain the inoculum, a museum culture of a bacterial strain stored on Schlegel agar medium at 5 °C was resuspended until a starting cell concentration of 0.1–0.2 g/L. The bacteria were cultured in Schlegel’s salt medium in periodic mode: during the first 25–35 h of growth, 0.7 g/L of a nitrogen source was added to the medium, which served as a limiting factor and stimulated the production of PHAs. The source of nitrogen was exhausted in the subsequent hours of growth.

During the growth of the bacteria, samples were taken periodically (every 24 h) to analyze the accumulation of biomass and polymers in the cells. The dynamics of the cell growth in the culture were recorded by the optical density of the bacterial suspension at a wavelength of λ = 440 nm (UNICO 2100, Dayton, NJ, USA). The cell concentration (i.e., the yield of bacterial biomass X, g/L) was assessed by the gravimetric method by drying a twice-washed biomass at 105 °C for 24 h, which was centrifuged at 6000 rpm.

### 2.2. PHA Analysis

To determine the intracellular content of the PHAs in bacterial cells and its monomer composition, dry cell biomass and native polymers extracted from the cells and purified to a homogeneous state were used. The extraction of PHAs from a biomass was carried out in two stages: (1) the actual extraction of the polymer with dichloromethane and the concentration of the resulting extract on a rotary evaporator; and (2) ethanol precipitation. To purify the obtained samples, the polymer was dissolved and reprecipitated several times.

The purity and composition of the polymer was determined by a chromatography of the methyl esters of fatty acids after methanolysis of the purified polymer samples using a 7890A chromatograph-mass spectrometer (Agilent Technologies, Santa Clara, CA, USA) equipped with a 5975C mass detector (Agilent Technologies, Santa Clara, CA, USA) [65]. Benzoic acid was used as an internal standard to determine the total intracellular PHAs [66]. 

### 2.3. PHA Properties

The molecular weight and molecular weight distribution of the PHAs were examined using a size-exclusion chromatograph (Agilent Technologies 1260 Infinity, Waldbronn, Germany) with a refractive index detector, and this was achieved using an Agilent PLgel Mixed-C column, where the weight average, M_w_, number average, M_n_ and polydispersity (Ð = M_w_/M_n_) were determined. The thermal properties of the polymer were analyzed using a DSC-1 differential scanning calorimeter (Mettler Toledo, Schwerzenbac, Switzerland). The melting points were determined from the exothermal peaks in the thermograms using the STARe software. Thermal degradation of the samples was investigated using a TGA2 thermal analysis system (Mettler Toledo, Schwerzenbac, Switzerland). The theoretical degree of crystallinity was calculated using the following formula:

C_x_ = (∆H_i_)/(∆H_0_), (1)

where ∆H_i_ is specific enthalpy of melting of the sample (J/g), and ∆H_0_ is the specific enthalpy of a melted 100% crystallized P(3HB) (146 J/g) [67]. The methods for analyzing the physicochemical properties of PHAs have been previously described in detail [59].

### 2.4. Statistical Analysis 

The statistical analysis of the results was performed by conventional methods using the standard software package of Microsoft Excel 2013 (Ver. 15.0.4420.1017). Each experiment was performed five times. The arithmetic means and standard deviations were found. To compare the groups, the Mann–Whitney test was used at the significance level of *p* ≤ 0.05.

## 3. Results and Discussion

### 3.1. Growth and PHA Synthesis by Bacteria C. necator B-10646 on Individual Saturated Fatty Acids

**Figure 1:**
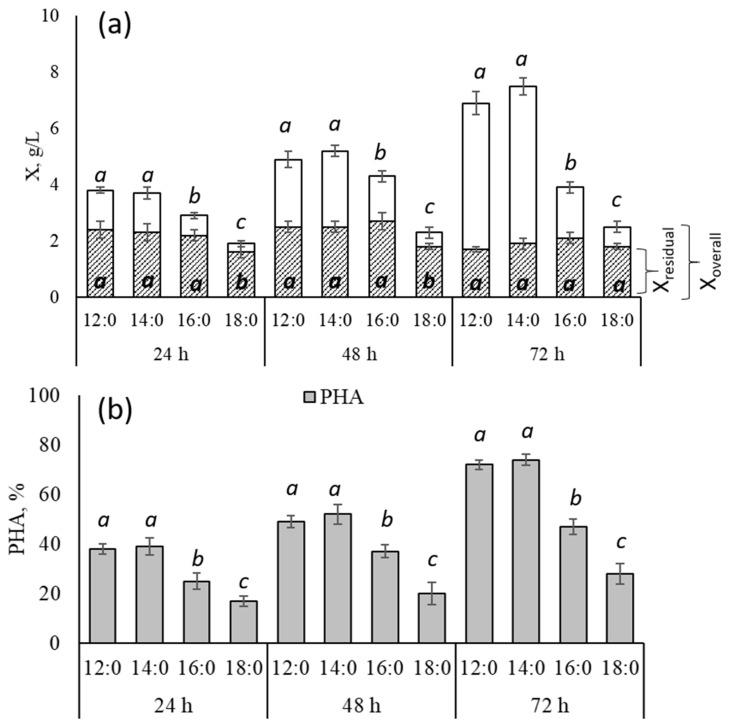
The yield of the overall (X, g/L) and residual bacterial biomass (X_res_, g/L) (**a**) and the intracellular PHA content (**b**) in the culture of the wild-type *C. necator* B-10646 strain during growth on the saturated FAs of various structures. The letters indicate the significance of the differences when comparing groups according to the Mann–Whitney test at a level of *p* ≤ 0.05, where identical letters indicate no significant differences.

The growth of *C. necator* B-10646 bacteria was conducted using one of following four saturated fatty acids as the sole carbon source: myristic (12:0), lauric (14:0), palmitic (16:0) and stearic (18:0) acids. These acids were studied for the first time. These fatty acids were chosen due to their wide distribution in vegetable oils and low-grade animal fats, which are currently being actively studied as a carbon substrate for the production of PHAs [29,68,69,70]. The results of assessing the ability of *C. necator* B-10646 bacteria to grow and synthesize polymers using individual saturated fatty acids as the sole carbon substrate are presented in Figure 1.

All the studied saturated fatty acids (FAs) were supported the growth of bacteria *C. necator* B-10646 and were accompanied by the synthesis and accumulation of the polymer in cells (Figure 1a). The highest yield of the bacterial biomass (X = 7.5 ± 0.4 g/L) was obtained when the bacteria were grown on myristic acid as the only carbon source; using lauric acid resulted in a slightly lower (6.7 ± 0.5 g/L) growth, but these differences were not statistically significant. In the aforementioned cases, the values of the residual bacterial biomass (X_res_) were 1.9 ± 0.1 and 1.7 ± 0.3 g/L, respectively, which also did not differ significantly. The lowest bacterial biomass yield (X = 2.5 ± 0.3 g/L, X_res_ = 1.8 ± 0.2 g/L) was recorded for stearic acid. The growth of the bacteria on palmitic acid was comparable to the results obtained on the myristic and lauric acid-grown FAs, but this was only found during the first 48 h of bacterial cultivation. Further, there was no increase in the X value; moreover, a decrease value was noted by 72 h, which indicates the death of some cells in the culture.

The intracellular content of the polymer in the culture of *C. necator* B-10646 during growth on the saturated FAs also varied (Figure 1b). The PHA content was comparable and the highest at 74 ± 2 and 72 ± 2% when bacteria were grown on myristic and lauric acids, respectively. The intracellular content of PHAs on palmitic acid was significantly lower (47 ± 3%); in the case of stearic acid, it was very low (28 ± 4%). The results of a comparative analysis of the production indicators of the *C. necator* B-10646 culture when bacteria were grown on the saturated FAs of various structures are presented in Table 2.

The highest values of all the studied indicators were obtained when myristic acid was used as the C-substrate. Thus, the productivity of the *C. necator* B-10646 culture calculated for the total growth period (72 h) in terms of the overall bacterial biomass (X, g/L) and polymer (PHAs) were 0.104 and 0.078 g/L·h, respectively; moreover, the economic coefficients (Y_X_ and Y_PHA_), were 0.83 and 0.62 g/g, respectively. The productivity indicators obtained for the bacterial growth on lauric acid were close to the results obtained from the growth on myristic acid, but the economic coefficients were significantly inferior. The biomass and polymer productivity decreased relative to myristic acid by two and three times, respectively, and the economic ratios also dropped significantly when the bacteria were grown on palmitic acid. The least productive was the process on stearic acid. When stearic acid was used as the only C-substrate, all of the calculated indicators decreased even more significantly than those obtained on palmitic acid (Table 2).

An analysis of the literature data showed that the yield of bacterial biomass and polymers when the bacteria of various species are grown on saturated fatty acids with a C-chain length from C12 to C18 also vary widely (Table 1). The wild-type and genetically modified *Pseudomonas* strains have been the most studied in terms of being grown on saturated FAs. This is in addition to the P(3HB) homopolymer PHA copolymers also have been synthesized containing medium-chain monomers of various structures. However, relatively low yields of bacterial biomass (from 1.0 to 5.5 g/L) and PHA yields of no more than 50% [71,72,73,74,75,76,77,78,79,80,81,82] have been obtained in most studies. This is also typical for other taxa, such as *Delftia* [49,53], *Burkholderia* sp. [50], *Klebsiella pneumonia* [45], *Bacillus cereus* [47] and *Rhodococcus pyridinivorans* [51]. A number of studies have described *Alcaligenes* (later *Cupriavidus*) strains as capable of accumulating up to 3.2 g/L of biomass that contain up to 55% of PHAs when they were grown on saturated the fatty acids of C12-C18 [44].

The yield of the biomass and PHAs obtained in the culture of *C. necator* B-10646 when using myristic acid exceeded the data available in the literature for most of the bacteria of various taxa [53,77,80], as well as for the closely related strains of *Alcaligenes* sp. AK 201 [44] and *C. necator* DSM 454 [48]. The quantitative indicators obtained in this paper when *C. necator* B-10646 was cultivated on lauric acid also exceed the data on the accumulation of bacterial biomass and PHAs in cultures of *A. hydrophila* and *A. salmocida* [45,46]. The published data on bacterial growth and PHA synthesis on palmitic and stearic fatty acids are limited. Furthermore, in general and in terms of production, the indicators regarding the most of all strains [48,50,78] have also been found to be inferior to the results obtained in this paper.

An important indicator of the efficiency of biotechnological processes that affects the economy of process is the completeness of the substrate being used, as well as, above all, carbon, the costs of which can reach 45–50% during the synthesis of PHAs [83]. It has been shown that the completeness of the studied saturated FAs when used by the *C. necator* B-10646, similar to the achieved production indicators (Table 2), varies significantly. At the same starting concentration of FAs in the nutrient medium (15.0 g/L), the amount of these substrates used by the bacterial culture during growth was 9.7, 9.0, 5.8 and 3.8 g/L, for lauric, myristic, palmitic and stearic FAs, respectively. The lauric and myristic fatty acids were completely utilized the most (by 64.7 and 60.0%, respectively). This indicator for palmitic and stearic fatty acids was almost two times lower and amounted to 38.7 and 25.3%, respectively (Table 2).

**Figure 2:**
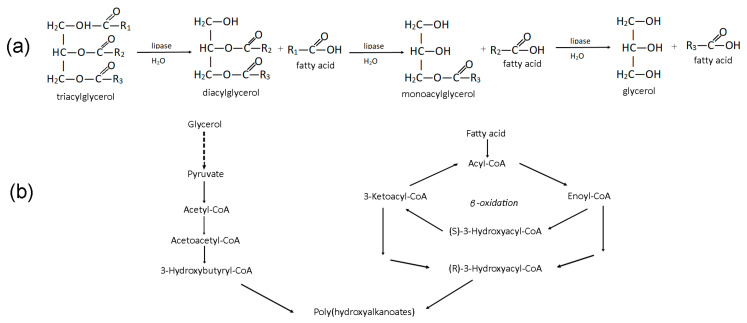
The scheme of the PHA synthesis from fatty acids: hydrolysis of the triacylglycerols to glycerol and free fatty acids (**a**) and pathways for the synthesis of PHAs from glycerol and fatty acids (**b**).

Thus, all of the saturated fatty acids studied were actively consumed and metabolized by *C. necator*. This is in contrast to the process on complex fatty substrates, where the fatty acids used were poor in terms of bacteria growth and accumulation in the culture. It is known that microbial metabolism depends on the activity of lipolytic enzymes, under the influence of which triacylglycerols (TAGs) are hydrolyzed to diacylglycerols (DAGs) and monoacylglycerols (MAGs) with the formation of the mixtures of glycerol and free fatty acids at the interface between lipids and water [84,85]. It is known that some FAs are freely transported into cells in a non-dissociated form as a result of nonionic diffusion [86]. In the cytoplasm of cells, fatty acids are metabolized via the *β*-oxidation pathway to form (*R*)-3-hydroxyacyl-CoA, which is a building block for PHA monomers [87]. The process of PHA synthesis in bacterial cells through the *β*-oxidation cycle of FAs, which are formed from TAG in complex fatty substrates, is shown in Figure 2.

The uneven consumption of FAs by bacteria is described in papers where the growth and synthesis of PHAs on vegetable and animal fats were studied [29,30,87]. The authors of these papers, via analyzing the process of lipid hydrolysis under the influence of microbial extracellular lipases, took into account the dynamics of the conversion of triacylglycerols into DAGs, MAGs and FFAs. It was then concluded that FAs with different carbon chain lengths, which are released from complex lipids, penetrate into cells and are metabolized differently. It is possible that the lipase activity of the studied *C. necator* B-10646 strain (determined at a level of 6.6–11.5 U/mL) is not active enough and does not ensure a complete hydrolysis of the lipid component of complex substrates, nor does it ensure the release of all acids into the medium, thus making them accessible to cells. This prompted us to investigate how the studied saturated FAs, when they are added simultaneously to the nutrient medium, are utilized in the mixtures.

### 3.2. Growth and PHA Synthesis by Bacteria C. necator B-10646 on Mixtures of Fatty Acids

The PHA synthesis on the mixtures of saturated FAs was studied for the first time. For the purposes of research, several variants of the mixtures of saturated fatty acids, differing in the set of fatty acids and their ratio (Table 3), were prepared.

The first mixture contained four saturated FAs that are characteristic for vegetable oils [88]: lauric, myristic, palmitic and stearic. The concentration of all acids was the same, i.e., 3.75 g/L, and the ratio used was 1.0/1.0/1.0/1.0 (at a total of 15.0 g/L). The second mixture contained three saturated acids, myristic, palmitic and stearic, which were found in the fat obtained from fish processing waste (in which lauric acid was not detected [35]). The total concentration of the three acids and their ratio in the medium were similar to the first mixture. The third mixture contained three FAs similar to Mixture 2, but the concentration of acids in the mixture was close to the content in the so-called sprat oil [42] and was 1.5, 11.7 and 1.8 g/L (total 15.0 g/L) for the myristic, palmitic and stearic acids, respectively, with an FA ratio of C14:0/C16:0/C18:0 = 1.0/7.8/1.2. 

**Figure 3:**
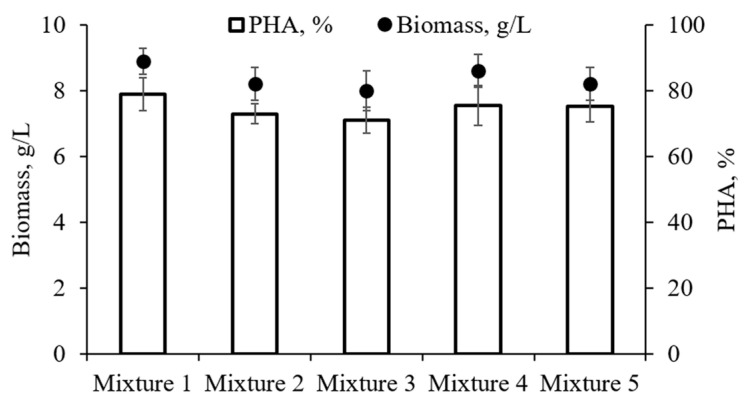
The bacterial biomass yield and intracellular PHA content in the culture of the wild-type *C. necator* B-10646 strain on the mixtures of fatty acids. The composition of the FA mixtures is detailed in Table 3.

The results of the growth and synthesis of the PHAs in the bacteria *C. necator* B-10646 strain using mixtures of saturated FAs as a carbon substrate are presented in Figure 3 and Table 4.

The bacterial biomass yields obtained by cultivating *C. necator* B-10646 on mixtures of fatty acids in most of the cases did not statistically differ significantly (X = 7.9–8.9 g/L when X_res_ was 1.9–2.4 g/L). Moreover, they slightly exceeded the results that were obtained when using individual fatty acids. Mixture 1, which contained the four studied acids, was the most effective. It provided a biomass and polymer productivity of 0.124 and 0.097 g/L*h, respectively. The use of Mixtures 2 and 3 ensured productivity in terms of the overall biomass and polymer at 0.110–0.114 and 0.076–0.083 g/L·h, respectively. The completeness of the utilization of the fatty acids in Mixtures 1 and 2 was close (92.7–94.9%); however, for Mixture 3, it was significantly lower (86.1%) than for Mixture 2. Meanwhile, it was noted that, in all cases, the completeness of the utilization of the fatty acids exceeded the results obtained with individual fatty acids (25.0–64.7%) (Table 4).

It is not possible to compare the results with published ones since no studies on the synthesis of PHAs on mixtures of saturated FAs can be found in the available literature.

**Figure 4:**
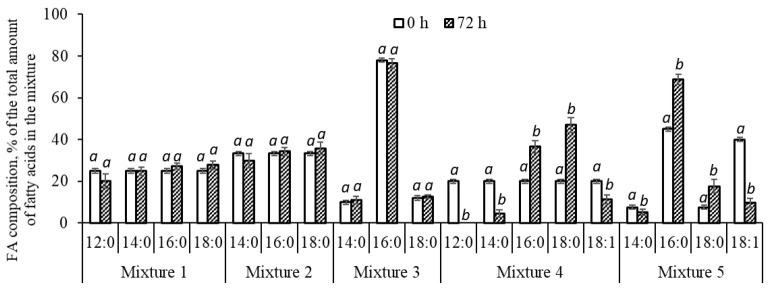
The ratio of the fatty acids in the initial nutrient medium and in the culture of the wild-type bacteria *C. necator* B-10646 strain at the end of the growth (72 h). The letters indicate the significance of differences when comparing the groups according to a Mann–Whitney test at the level of *p* ≤ 0.05, where identical letters indicate no significant differences.

The utilization of individual fatty acids from the studied mixtures is illustrated in Figure 4. A comparison of the initial proportions of the FAs in the nutrient medium with the residual ones in the culture showed no changes. This is an indicator of the uniform utilization of fatty acids by the bacteria during growth on the studied mixtures of FAs, which is in contrast to the uneven consumption of these FAs from complex fat-containing substrates.

The revealed effects of the uneven consumption of FAs by bacteria when grown on substrates of complex composition are, apparently, because natural fat-containing sources contain a wide range of fatty acids of various structures, including saturated and unsaturated acids, mono- and polyenoic, branched, etc. Seemingly, the process of FA transport and the affinity for bacteria as a growth substrate are different. It has been shown that bacteria primarily utilize polyenoic fatty acids, while monoenoic and saturated fatty acids are poorly or not metabolized at all, so their content in the residual substrate increases [41,42,89]. It is also possible that there is competition between FAs for the transporter in the processes of the active transport of fatty acids into cells.

Therefore, it was considered appropriate to study the growth of bacteria and the dynamics of fatty acid consumption if the composition of the mixtures became more complex. FA mixtures were prepared and studied (Table 3), to which oleic acid (unsaturated fatty acid) was added. Oleic acid, as a rule, dominates in the composition of natural fats of various origins and can account for 20–25% or more in relation to the number of FAs. Mixture 4 was based on Mixture 1, i.e., oleic acid was added to four unsaturated FAs, the ratio of all FAs was the same and their total concentration was 20 g/L. Mixture 5 contained a double concentration of oleic acid, but the shortest acid, lauric acid, was excluded from it, that is, palmitic and oleic fatty acids were found to dominate in this mixture. This corresponds to the FA content in sprat oil [42].

The *C. necator* B-10646 cultivation in Mixture 4 (which contained, in addition to saturated FAs, unsaturated oleic acid in equal parts) did not significantly affect the production indicators of the culture (Table 4), which, in terms of biomass and PHA yields, were comparable to the results obtained on FA mixtures without oleic acid. At the same time, it was revealed that the consumption of fatty acids by bacteria from the four- and five-component mixtures with oleic acid changes (Figure 4). The presence of oleic acid in both variants negatively affected the consumption of palmitic and stearic fatty acids by the bacteria, the proportion of which among the residual FAs in the culture increased but did not affect the utilization of lauric and myristic acids. Oleic acid, in contrast to the growth of this strain on complex fat-containing substrates (as we previously showed [42]), was consumed by bacteria. Its concentration in the culture compared to the initial concentration in the nutrient medium significantly decreased from 4.0 to 2.3 g/L in Mixture 4 and, more significantly, from 8.0 to 1.9 g/L in Mixture 5.

The obtained results showing a decrease in the consumption of individual saturated fatty acids by bacteria in the presence of oleic acid, in our opinion, may indicate support for the assumption of competitive relationships between FAs in the processes of their transport into the cell. According to the study of [90], the transport of free fatty acids into the cell, depending on the length of the carbon chain, can be passive or active. Therefore, short-chain (C4–C6) and medium-chain fatty acids (C7–C11) can enter the cell by free diffusion, while longer fatty acids (>C12) require specialized transporters. It has been established that, in the cells of Gram-negative bacteria, the FA transport protein is FadL, which is located on the outer side of the cell membrane [91]. Long-chain fatty acids penetrate the outer membrane through FadL, which then pass through the periplasmic space and enter the inner membrane. It is believed that, after being transported across the membrane, fatty acids enter the periplasmic space. This is where, changing their orientation, they penetrate the inner membrane layer and are activated by the cytosolic acyl-CoA synthase FadD after that enter the *β*-oxidation cycle [90]. It can be assumed that a similar mechanism of FA transport is also implemented in most Gram-negative bacteria, including representatives of *C. necator.* Due to the fact that the issue of the transport of fatty acids from the periplasmic space through the inner cell membrane has not yet been fully studied, the reason for the uneven utilization of fatty acids may lie precisely in this aspect of FA transport into the cell. However, this issue requires additional and special research.

### 3.3. The Composition and Physicochemical Properties of PHAs Synthesized by C. necator B-10646 on Individual Fatty Acids and Their Mixtures

PHAs are a family of polymers with a different set and ratio of monomers, so their properties vary significantly. The composition of PHAs that are synthesized depends on many factors and, above all, on the physiological and biochemical specificity of the producer strains and carbon nutrition conditions. The published data on the properties of PHAs synthesized by the bacteria of various taxa on various fatty acids as the sole carbon source are very limited (Table 1). The information in Table 1 shows that the available data on the properties of PHAs synthesized on FAs are extremely limited. At the same time, the need and importance for studying the properties of PHAs that are synthesized on new substrates or new strains is due to the fact that the value of the samples is determined not only by the yield of the polymer, but also by the manufacturability of these polymers, that is, the possibility of processing and obtaining products from them. This is determined by the physicochemical properties of polymers, which primarily involve molecular weight and temperature characteristics. These situations are possible when a particular producer is capable of PHA synthesis; however, properties such as, for example, the degree of polymerizability or thermal behavior are such that the processing and obtaining of products from these polymers using accessible methods is difficult or impossible.

In the presented paper, the properties of PHAs synthesized on saturated fatty acids was studied for the first time. At the same time, the composition and properties of polymers synthesized by *C. necator* B-10646 were studied both on individual saturated FAs and on their mixtures (Table 5).

**Figure 5:**
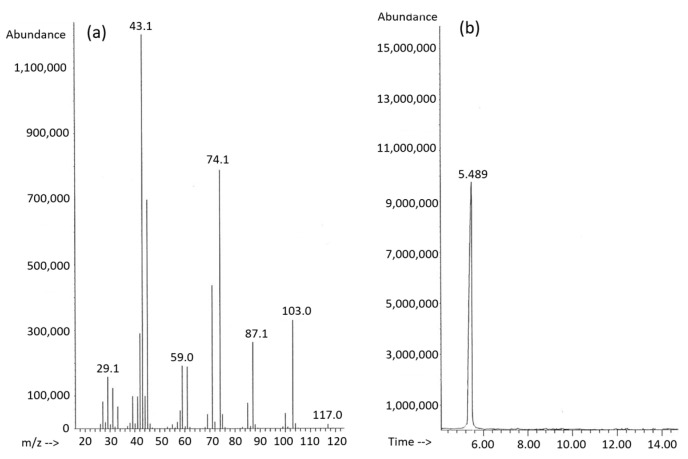
The mass spectrum (**a**) and ion chromatogram (**b**) of the P(3HB) sample synthesized by the wild-type *C. necator* B-10646 strain on saturated FAs. The retention time of the 3HB unit is 5.489 min.

According to gas chromatography data, all of the synthesized PHA samples represent a poly(3-hydroxybutyrate) homopolymer regardless of whether fatty acids were supplied as a monosubstrate or as a part of mixtures. As an example, Figure 5 shows an ion chromatogram and mass spectrum, wherein the monomeric composition of P(3HB) synthesized by *C. necator* B-10646 using lauric acid is illustrated.

**Figure 6:**
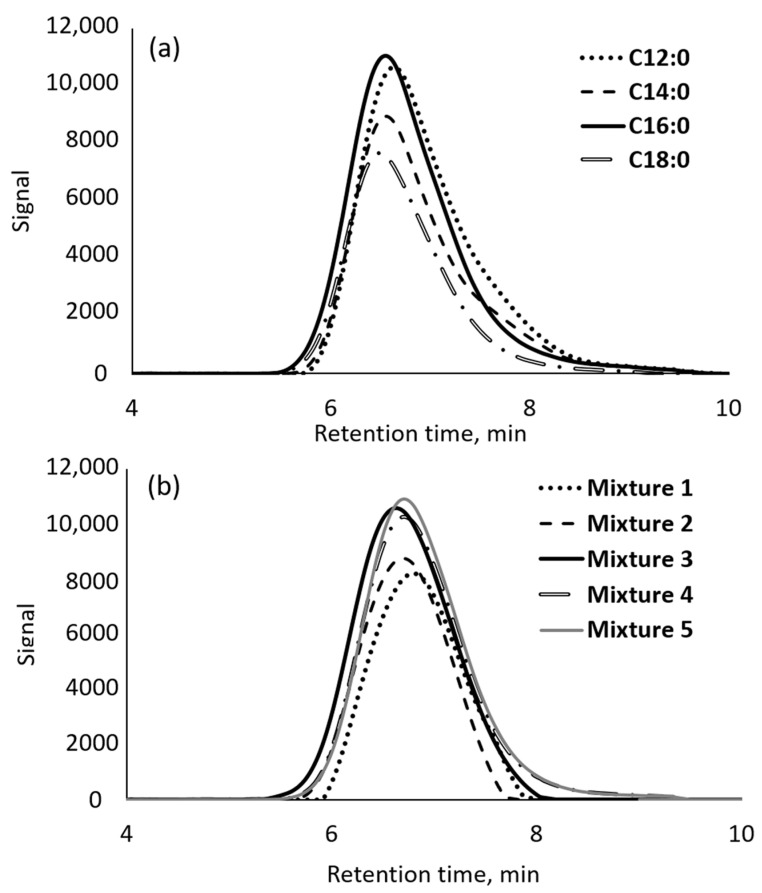
GPC chromatogram of the P(3HB) samples synthesized by the wild-type *C. necator* B-10646 strain on individual saturated FAs (**a**) and on the mixtures of FAs (**b**). The composition of FA mixtures corresponds to Table 3.

The molecular weight characteristics of the P(3HB) samples synthesized by *C. necator* B-10646 on the individual saturated FAs and mixtures of FAs differed (Figure 6). One can note a tendency for the average molecular weight of the polymer to increase and for the polydispersity to decrease with the lengthening of the carbon chain of the studied saturated FAs as the sole C-substrate.

The average molecular weight (M_w_) of the P(3HB) samples increased almost 1.5 times—from 305.5 to 447.1 kDa—with an increase in the length of the carbon chain in the fatty acids (from 12 to 18 carbon atoms in the lauric and stearic fatty acids, respectively) against the background of a decrease, from 3.74 to 2.88, in the polydispersity (Đ). The molecular weight (M_w_) of the sample obtained by *C. necator* B-10646 cultivation on the FA Mixture 1 was somewhat lower than this indicator when individual fatty acids were used, whereby it amounted to 289.3 kDa with a polydispersity value of 2.72. When bacteria were grown on the FA Mixture 3, the P(3HB) samples had a slightly increased molecular weight compared to individual fatty acids (M_w_ = 464.5 kDa at Đ = 3.48). The polymer samples synthesized on all other mixtures (FA mixtures 2 and 4–5) were comparable in their molecular weight characteristics to those obtained using individual fatty acids.

An analysis of publications showed that the effect of fatty acids on the molecular weight characteristics of PHAs was the most studied issue. The results of a study of the average molecular weight of P(3HB) synthesized by *C. necator* B-10646 using lauric fatty acid were found to be consistent with the results obtained in [44], where the M_w_ value of the polymer samples synthesized by the *Alcaligenes* sp. AK 201 strain on lauric acid was 304 kDa. When lauric acid was replaced with fatty acids that have a longer C-chain length, an increase in M_w_ values was found, and these changes were more pronounced [44]. The average molecular weight of the polymer increased to 1442 kDa when the bacteria was grown on myristic and palmitic acids, and it was somewhat less, to 986 kDa, on stearic acid [44].

**Figure 7:**
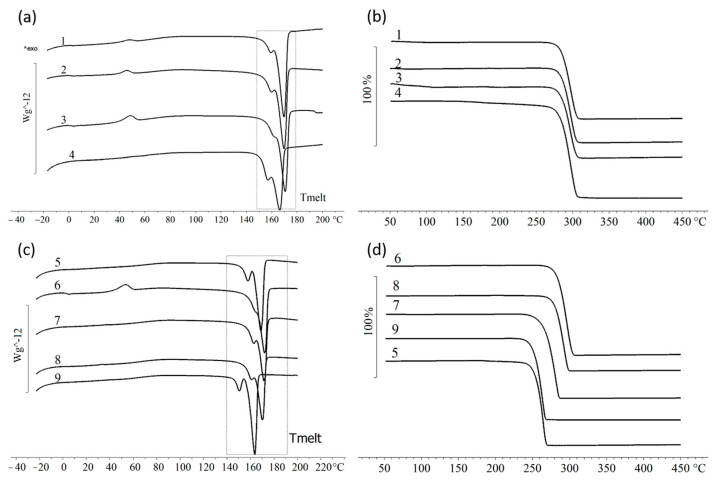
The temperature characteristics of P(3HB) samples synthesized by the wild-type *C. necator* B-10646 strain on individual saturated FA–DSC curves (**a**) and thermal stability (TGA) (**b**). The temperature characteristics of the P(3HB) samples synthesized on FA mixture–DSC curves (**c**) and thermal stability (TGA) (**d**). The numbering of polymer samples corresponds to Table 5.

The temperature properties of the P(3HB) samples synthesized on individual saturated FAs and mixtures of FAs are presented in Table 5 and Figure 7.

It was shown that all of the samples of polymers synthesized on the studied saturated fatty acids had a similar melting point (T_melt_), 166–170 °C, which is typical for P(3HB). The melting peak in the thermograms (Figure 7) was found to be narrow and had a small shoulder on the low-temperature side. The samples synthesized on lauric, myristic and palmitic acids were characterized by the presence of two crystallization peaks: the first peak was when the samples were cooled (60–67 °C), and the second peak was when they were reheated (45–48 °C). The samples of polymers synthesized using the fatty acids studied demonstrated good thermal stability. The gap between the melting point and the thermal degradation temperature (T_degr_) was 114–118 °C, and the average thermal degradation temperature of the samples was 284 ± 1 °C.

The thermograms of the P(3HB) samples synthesized on the mixtures of fatty acids were characterized by narrow melting peaks. The samples synthesized on Mixtures 1 and 5 had two melting peaks; for the other samples, this peak degenerated into a small shoulder at the main melting peak on the low-temperature side. For these polymer samples (which were obtained when Mixtures 1 and 5 were used), the lowest melting point values were recorded at 157.4 and 168.6 °C and 150.3 and 163.1 °C accordingly. For the samples obtained on Mixtures 2–4, the melting point was 170–171 °C, which is close to the melting point of the polymer samples synthesized on individual fatty acids.

The samples of the polymers synthesized on mixtures of fatty acids demonstrated varying thermal stability, and these were slightly different from the samples obtained on separate fatty acids. The highest temperature of thermal degradation was recorded for those polymers obtained using Mixture 2 (295.7 °C). The lowest temperature of thermal degradation and, consequently, the lowest thermostability was characteristic of the samples obtained using Mixtures 1, 3 and 5. In those cases, the T_degr_ values were 267.6, 264.5 and 252.4 °C, respectively.

In the available literature, the data on the temperature characteristics of P(3HB) that were obtained on individual fatty acids are limited. Most of the data published relate to multicomponent copolymers that are synthesized by representatives of *Pseudomonas*, where the T_melt_ lies in the range of 52–77 °C [46,47,48,49,50,51,71,72,73,74,75,76,77]. The results obtained in this work are close to the temperature characteristics of the P(3HB) synthesized by the *D. tsuruhatensis* Bet002 strain on individual fatty acids (T_melt_ 173.2–177.4 °C; T_degr_ 289.8–391.8 °C) [49].

No data on the degree of the crystallinity of PHAs synthesized on saturated fatty acids with a carbon chain length of 12–18 carbon atoms have been found in the available literature. The degree of the crystallinity of the P(3HB) samples synthesized by the *C. necator* B-10646 strain, except for the sample that was obtained using Mixture 1, was determined from thermograms, and it was found to be close and ranged from 47 to 57%. This value is below the known data, where the C_x_ of a P(3HB) is usually determined higher (from 60% and above). The lowest degree of crystallinity (39%) was recorded for the polymer sample that was obtained from Mixture 1 (Table 5), which is unusual for a P(3HB) homopolymer.

The novelty and significance of the results obtained is due, firstly, to the new data on the properties of PHAs, which were synthesized on a little-studied carbon substrate, i.e., saturated FAs. The study showed that, in general, the basic properties (i.e., the molecular mass and temperature characteristics) generally correspond to the data, and they are characteristic of the polymer samples synthesized on many other generally used C-substrates. The revealed reduced values of the degree of crystallinity can be regarded as a positive point since a decrease in crystallinity makes a PHA sample more technological. Secondly, the results obtained allow us to consider saturated fatty acids as a C-substrate for the synthesis of PHAs by the highly productive wild-type *C. necator* B-10646 strain, and they also indicates an increase in the completeness of the use of complex fat-containing waste, of which saturated acids, as a rule, are poorly utilized by bacteria.

## 4. Conclusions

It was shown that saturated FAs with different C-chain lengths (C12, C14, C16 and C18), individually and in mixtures, ensure the growth and synthesis of PHAs by the wild-type *Cupriavidus necator* B-10646 strain. The length of the C-chain of SFAs influences the overall yield of the biomass and PHAs, which are two times higher when bacteria are grown on C12 and C14 FAs compared to longer C16 and C18 acids. On all of the types of SFAs, the bacteria synthesized poly(3-hydroxybutyrate) with typical molecular weight and temperature characteristics but reduced crystallinity. The ability of bacteria to assimilate SFAs opens up the possibility of attracting the synthesis of PHAs to complex fat-containing substrates, including waste.

## Figures and Tables

**Table 1:** Published data on the P(3HB) synthesis by various wild-type producers during growth on different fatty acids as a carbon source.

Strain	X, g/L	P(3HB), %	M_w_, kDa	Đ	T_melt_, °C	T_degr_, °C	C_x_, %	Reference
Lauric acid								
*Alcaligenes* sp. AK 201	~2.4	40%	304	1.9	102	-	-	[44]
*Aeromonas hydrophila*	2.7–4.3	19.4–41.1	-	-	-	-	-	[45,46]
*Aeromonas salmonicida* 741	2.8–3.8	13.3–30.0 *	-	-	-	-	-	[45]
*Klebsiella pneumoniae* ZMd31	0.6–0.7	11.0–18.3 *	-	-	-	-	-	[45]
*Burkholderia* sp.	1.4–2.1	8–69	-	-	-	-	-	[45]
*Bacillus cereus* SPV	0.51	61.81	-	-	-	-	-	[47]
Myristic acid								
*Alcaligenes* sp. AK 201	~2.9	55%	1416	3.1	106	-	-	[44]
*C. necator* DSM 545	1.43	-	-	-	-	-	-	[48]
*D. tsuruhatensis* Bet002	-	76.7	131	1.1	173.2	289.8	-	[49]
*Burkholderia* sp.	1.1–1.9	1–49	-	-	-	-	-	[50]
Palmitic acid								
*Alcaligenes* sp. AK 201	~3.2	55%	1442	2.5	108	-	-	[44]
*C. necator* DSM 545	1.97	-	-	-	-	-	-	[48]
*D. tsuruhatensis* Bet002	-	53.8	166	1.5	175.7	302.9	-	[49]
*Burkholderia* sp.	0.6–1.5	tr-9	-	-	-	-	-	[50]
*Rhodococcus pyridinivorans* KY703220	1.435 (OD)	40	-	-	-	-	-	[51]
Stearic acid								
*Alcaligenes* sp. AK 201	~2.3	30%	986	1.9	116	-	-	[44]
*C. necator* DSM 545	2.11	-	-	-	-	-	-	[48]
*D. tsuruhatensis* Bet002	-	45.0	188	1.8	177.4	391.8	-	[49]
*Burkholderia* sp.	0.5–1.0	tr-1	-	-	-	-	-	[50]

* The data on the PHA composition are not reported.

**Table 2:** The production indicators in the culture of the wild-type *C. necator* B-10646 strain on the saturated FAs of various structures.

Fatty Acid (FA)	X, g/L	PHAs, g/L	X_res_, g/L	PHAs, %	Y_X_, g/g	Y_PHA_, g/g	Biomass Productivity		PHA Productivity, P_PHA_, g/L·h	Degree of Use of FAs, %
							P_X_, g/L·h	P_Xres_, g/L·h		
Lauric C12:0	6.7 ^a^	5.0 ^a^	1.7 ^a^	75 ^a^	0.69 ^a^	0.52 ^a^	0.093 ^a^	0.024 ^a^	0.069 ^a^	64.7 ^a^
Myristic C14:0	7.5 ^a^	5.6 ^a^	1.9 ^a^	74 ^a^	0.83 ^b^	0.62 ^a^	0.104 ^a^	0.026 ^a^	0.078 ^a^	60.0 ^a^
Palmitic C16:0	3.9 ^b^	1.8 ^b^	2.1 ^a^	47 ^b^	0.67 ^a^	0.31 ^b^	0.054 ^b^	0.029 ^a^	0.025 ^b^	38.7 ^b^
Stearic C18:0	2.5 ^c^	0.7 ^c^	1.8 ^a^	28 ^c^	0.66 ^a^	0.18 ^c^	0.035 ^c^	0.025 ^a^	0.010 ^c^	25.3 ^c^

The letters indicate the significance of the differences when comparing groups according to the Mann–Whitney test at the level of *p* ≤ 0.05, where identical letters indicate no significant differences.

**Table 3:** Composition of the FA mixtures as a carbon substrate for the growth of the wild-type *C. necator* B-10646 strain.

Mixture Number	FA Composition in the Mixture	FA Concentration, g/L	Total of FA, g/L	FA Ratio
1	Lauric C12:0	3.75	15.0	C12:0/C14:0/C16:0/C18:0 = 1.0/1.0/1.0/1.0
	Myristic C14:0	3.75		
	Palmitic C16:0	3.75		
	Stearic C18:0	3.75		
2	Myristic C14:0	5.0	15.0	C14:0/C16:0/C18:0 = 1.0/1.0/1.0
	Palmitic C16:0	5.0		
	Stearic C18:0	5.0		
3	Myristic C14:0	1.5	15.0	C14:0/C16:0/C18:0 = 1.0/7.8/1.2
	Palmitic C16:0	11.7		
	Stearic C18:0	1.8		
4	Lauric C12:0	4.0	20.0	C12:0/C14:0/C16:0/C18:0/C18:1ω9 = 1.0/1.0/1.0/1.0/1.0
	Myristic C14:0	4.0		
	Palmitic C16:0	4.0		
	Stearic C18:0	4.0		
	Oleic C18:1ω9	4.0		
5	Myristic C14:0	1.5	20.0	C14:0/C16:0/C18:0/C18:1ω9 = 1.0/6.0/1.0/5.3
	Palmitic C16:0	9.0		
	Stearic C18:0	1.5		
	Oleic C18:1ω9	8.0		

**Table 4:** The production indicators of the wild-type *C. necator* B-10646 strain on mixtures of FAs of various content.

FA Mixture Number	X, g/L	PHAs, g/L	PHAs, %	X_res_, g/L	Y_X_, g/g	Y_PHA_, g/g	Biomass Productivity		PHA Productivity, P_PHA_, g/L·h	Degree of the Use of FAs, %
							P_X_, g/L·h	P_Xres_, g/L·h		
Saturated FAs										
1	8.9 ^a^	7.0 ^a^	79 ^a^	1.9 ^a^	0.63 ^ab^	0.49 ^ac^	0.124 ^a^	0.026 ^a^	0.097 ^a^	94.9 ^ab^
2	8.2 ^ab^	6.0 ^b^	73 ^a^	2.2 ^a^	0.59 ^a^	0.43 ^b^	0.114 ^ab^	0.031 ^a^	0.083 ^b^	92.7 ^a^
3	8.0 ^b^	5.7 ^b^	71 ^a^	2.4 ^a^	0.62 ^ab^	0.44 ^abc^	0.111 ^b^	0.033 ^a^	0.079 ^b^	86.1 ^bcd^
Saturated FAs + Oleic acid										
4	8.6 ^ab^	6.5 ^ab^	76 ^a^	2.1 ^a^	0.69 ^b^	0.52 ^ac^	0.119 ^ab^	0.029 ^a^	0.090 ^ab^	83.1 ^c^
5	8.2 ^ab^	6.1 ^ab^	75 ^a^	2.1 ^a^	0.61 ^ab^	0.45 ^abc^	0.114 ^ab^	0.029 ^a^	0.085 ^ab^	90.4 ^ad^

The composition of the FA mixtures corresponds to Table 3. The letters indicate the significance of differences when comparing groups according to the Mann–Whitney test at the level of *p* ≤ 0.05, where identical letters indicate no significant differences.

**Table 5:** The properties of the P(3HB) samples synthesized by the wild-type *C. necator* B-10646 strain on the individual saturated FAs and mixtures of FAs.

P(3HB) Sample Number	Substrate	M_w_, kDa	Đ	C_x_, %	T_melt_, °C	H_melt_, J/g	T_degr_, °C	T_g_, °C	T_cryst_, °C
Saturated FAs									
1	Lauric (12:0)	305.5	3.74	49.7	169.3	72.6	127.1 (19.5%) 284.7	-	65.2 47.5
2	Myristic (14:0)	364.9	3.71	52.8	169.2	77.1	113.0 (19.4%) 284.9	1.8	67.4 45.5
3	Palmitic (16:0)	423.7	3.46	50.7	170.1	74.1	113.0 (21.4%) 284.7	2.0	60.7 48.3
4	Stearic (18:0)	447.1	2.88	47.5	166.0	69.3	282.9	-	53.2
FA Mixture *									
5	Mixture 1	289.3	2.72	39.2	157.4 168.6	7.9 49.3	92.4 (14.9%) 264.5	-	74.1
6	Mixture 2	333.4	2.74	53.2	171.5	77.7	127.5 (9.2%) 295.7	3.0	62.1
7	Mixture 3	464.5	3.48	56.0	171.0	81.8	115.3 (17%) 267.6	-	66.0
8	Mixture 4	325.0	3.22	57.1	169.8	83.4	111.3 (22.8%) 281.1	-	65.3
9	Mixture 5	315.5	3.32	49.4	150.3 163.1	10.4 61.7	77.1 (3.6%) 138.5 (8.1%) 252.4	-	64.4

* The composition of FA mixtures corresponds to Table 3.

## Data Availability

All the data are available in the paper.
